# Comparison of Ventricular Inducibility with Late Gadolinium Enhancement and Myocardial Inflammation in Endomyocardial Biopsy in Patients with Dilated Cardiomyopathy

**DOI:** 10.1371/journal.pone.0167616

**Published:** 2016-12-08

**Authors:** Karin A. L. Mueller, Christian Heck, David Heinzmann, Johannes Schwille, Karin Klingel, Reinhard Kandolf, Ulrich Kramer, Michael Gramlich, Tobias Geisler, Meinrad P. Gawaz, Juergen Schreieck, Peter Seizer

**Affiliations:** 1 Medizinische Klinik III, Kardiologie und Kreislauferkrankungen, Universitaetsklinikum der Eberhard-Karls Universitaet, Tuebingen, Germany; 2 Abteilung für Molekulare Pathologie, Universitaetsklinikum der Eberhard-Karls Universitaet, Tuebingen, Germany; 3 Institut für Radiologie, Universitaetsklinikum der Eberhard-Karls Universitaet, Tuebingen, Germany; Cedars-Sinai Medical Center, UNITED STATES

## Abstract

**Background:**

Risk stratification of patients with non-ischemic dilated cardiomyopathy remains a matter of debate in the era of device implantation.

**Objective:**

We investigated associations between histopathological findings, contrast-enhanced cardiac MRI and the inducibility of ventricular tachycardia (VT) or fibrillation (VF) in programmed ventricular stimulation.

**Methods:**

56 patients with impaired left ventricular ejection fraction (LVEF≤50%, mean 36.6±10.5%) due to non-ischemic dilated cardiomyopathy underwent cardiac MRI, programmed ventricular stimulation, and endomyocardial biopsy and were retrospectively investigated. Inducibility was defined as sustained mono- or polymorphic VT or unstable VT/VF requiring cardioversion/defibrillation. Primary study endpoint was defined as the occurrence of hemodynamically relevant VT/VF and/or adequate ICD-therapy during follow-up.

**Results:**

Endomyocardial biopsy detected cardiac fibrosis in 18 (32.1%) patients. Cardiac MRI revealed 35 (62.5%) patients with positive late gadolinium enhancement. VT/VF was induced in ten (17.9%) patients during programmed ventricular stimulation. Monomorphic VT was inducible in 70%, while 20% of patients showed polymorphic VT. One patient (10%) presented with VF. Inducibility correlated significantly with the presence of positive late gadolinium enhancement in cardiac MRI (p<0.01). We could not find a significant association between inducibility and the degree of cardiac inflammation and fibrosis in non-site directed routine right ventricular endomyocardial biopsy. During a mean follow-up of 2.6 years, nine (16.1%) patients reached the primary endpoint. Monomorphic VTs were found in 66.7% patients and were terminated by antitachycardia pacing therapy. One patient with polymorphic VT and two patients with VF received adequate therapy by an ICD-shock. However, inducibility did not correlate with the occurrence of endpoints.

**Conclusion:**

Inducibilty during programmed ventricular stimulation is associated with positive late gadolinium enhancement in cardiac MRI of patients with non-ischemic dilated cardiomyopathy. The presence of myocardial fibrosis or inflammation in undirected endomyocardial biopsy does not seem to be sufficient to predict future ventricular arrhythmias.

## Introduction

Non-ischemic dilated cardiomyopathy (NIDCM) is a common cause of congestive heart failure (HF) with the subsequent need for intensified pharmacological treatment, implantation of cardioverter-defibrillators (ICDs), therapy of ventricular tachycardias (VTs), or even heart transplantation. Despite improved therapeutic approaches during the last decades, NIDCM is still associated with significant morbidity, premature mortality and therefore poor prognosis [1]. Several trials have shown that patients’ outcome benefits significantly from device implantation by reducing the incidence of sudden cardiac death in primary and secondary prevention but at considerable costs [2] and risks of complications associated with the implantation [3]. The decision, when to provide appropriate primary prevention ICD implantation in NIDCM remains a matter of debate [3, 4]. Current guidelines for ICD implantation in NIDCM are based on left ventricular ejection fraction (LVEF) and New York Heart Association (NYHA) functional class [5]. Yet, there is no evidence that these selection criteria are sufficient to identify patients, who will benefit most from ICD implantation [4–6]. For instance, there is a large number of NIDCM patients with only mild to moderate impaired LVEF, who die of sudden cardiac death. In contrary, up to 80% of NIDCM patients with an ICD and LVEF <35% will not experience device activation during their course of the disease. These findings suggest, that impaired LVEF alone is not a sufficient criterion for prognostic stratification regarding risk of sudden cardiac death and the indication for ICD implantation in NIDCM patients [6, 7]. While no other single marker has been proven to be as robust as LVEF [7], the combination of LVEF with other clinical or laboratory findings might further improve risk assessment in high- and low-risk patients [6–10]. Therefore, it is inevitable to intensify risk assessment and to identify patients at risk for proceeding HF requiring hospitalization and at risk for sudden cardiac death at an early stage of the disease [5].

There is a growing body of evidence, that the presence of myocardial fibrosis is associated with the occurrence of clinically relevant VTs and treatment failure [11]. The prognosis of patients with NIDCM has been linked to the degree of inflammation and the presence and amount of fibrosis within the myocardium [12–14]. Therefore, endomyocardial biopsies (EMB) are of great clinical importance for histopathological and immunohistochemical detection of cardiac fibrosis and infiltrating inflammatory cells [13].

Furthermore, several prospective studies have recently shown that the presence of myocardial fibrosis identified by contrast-enhanced cardiac magnetic resonance imaging (MRI) is associated with cardiovascular morbidity, overall mortality and likelihood of adequate ICD-therapy in patients with NIDCM [8, 12, 15]. Approximately 30% of patients with NIDCM show midwall fibrosis. These studies confirmed a low arrhythmic risk in NIDCM patients, who showed negative late gadolinium enhancement (LGE) in their MRI [10], while presence of positive myocardial LGE predicted adequate therapy by ICDs in these patients [16].

Along with other diagnostic tools, programmed ventricular stimulation (PVS) might also contribute to an improved risk assessment in patients with NIDCM. PVS demonstrated clinical relevance in the risk stratification of patients with ischemic cardiomyopathy [17, 18]. However, the benefit and relevance of invasive electrophysiological studies in patients with NIDCM remain a matter of debate. Recent studies on the electroanatomical characterization of LGE in a patient collective with an impaired LVEF≤50% due to NIDCM, detected a correlation between the occurrence of ventricular arrhythmias and the presence and area of LGE [19]. The presence and localization of LGE in cardiac MRI correlated significantly with functionally relevant findings in electroanatomical voltage mapping. In this study, presence of LGE predicted higher inducibility of VTs during PVS in patients with non-ischemic congestive HF [20].

To this point, there are only a few small studies investigating the role of invasive electrophysiology in patients with NIDCM. However, these reported collectives have only been characterized by their reduced LVEF and the exclusion of relevant coronary artery disease, while other non-invasive and invasive diagnostic tools, e.g. biopsy results, were not included [17, 21–23]. To our knowledge, the impact of endomyocardial biopsy (EMB) and its significance in the risk assessment of NIDCM patients has not been described yet.

Therefore, we evaluated the correlation of positive LGE in cardiac MRI and the inflammatory degree and presence of fibrosis in EMB with the inducibility of ventricular arrhythmias during PVS in patients with impaired LVF due to NIDCM. To our knowledge, the present study is the first of its kind to compare diagnostic findings of histopathology, immunohistochemistry, and cardiac MRI with results in PVS, in particular the inducibility of VT or ventricular fibrillation (VF).

## Materials and Methods

### Study design, patient collective and assessment of clinical risk factors

A retrospective analysis was performed between June 2009 and July 2013 including 56 patients with impaired LVEF≤50% due to NIDCM, who underwent clinically indicated EMB. All patients were admitted to the University Hospital Tuebingen due to impaired LVEF ≤50%. Indications for EMB were based on clinical indications as described before [1, 5, 24]. All invasive procedures were done in recompensated, stable patients. PVS was performed based on individualized criterions in patients who had a history suggesting ventricular arrhythmias (syncopes, presyncopes, non-sustained VTs, palpitations). Patients with secondary cardiomyopathy due to underlying diseases like systemic sclerosis, sarcoidosis, storage disease or other secondary cardiomyopathies were excluded. EMB and electrophysiological evaluation by PVS were performed within one week of hospital admittance in stable patients. Cardiomyopathies were defined according to proposed classification criteria [25].

All patients underwent clinical evaluation, laboratory testing, transthoracic echocardiography (TTE), left heart catheterization, EMB and PVS at study entry. All patients were medically treated according to current guidelines depending on degree of HF symptoms and LVF status [1, 5].

Clinical risk factors at study entry included age, gender, body mass index (BMI), NYHA functional class, and concomitant medication. Troponin I (TnI, normal value <0.03 μg/l), b-type natriuretic peptide (BNP, normal value >100 ng/l), creatine kinase (CK, normal value < 190U/l) and C-reactive protein (CRP) (normal value <0.5mg/dl) were assessed as laboratory markers. Echocardiographic parameters included LVEF, left ventricular enddiastolic diameter (LVEDD), right ventricular function, and systolic pulmonary arterial pressure (PAPsys). LVEF was estimated by echocardiography (iE33, Philips Medical Systems) using the modified Simpson rule with images obtained from apical 4- and 2-chamber views. Significant coronary artery disease (> 50% diameter luminal stenosis of two or more coronary vessels or left main or proximal left anterior descending coronary artery stenosis > 50%) was ruled out by coronary angiography in all patients.

The study conformed to the principles outlined in the Declaration of Helsinki, written informed consent was obtained from all patients. The study was approved by the local ethical committee of the Eberhard Karl University Tuebingen (95/2009BO1).

### Study endpoints and follow-up

56 patients enrolled in the study presented in our outpatient clinic for clinical follow-up every 3 to 6 months. 9 patients, who were not seen in the outpatient clinic, underwent a telephone interview 4 times during follow up to determine the occurrence of the study endpoint. Clinical charts were reviewed and the primary care physician was contacted on patient’s status, to evaluate medication and clinical symptoms of HF. None of the patients were lost in follow-up. Primary study endpoint was defined as the occurrence of hemodynamically relevant VT/VF or adequate ICD-therapy (antitachycardia pacing (ATP) or shock) within a mean follow-up period of 2.6 years.

### Endomyocardial biopsy, histopathological and immunohistochemical analysis

Biopsy sample site was the septum of the right ventricle in all patients and at least six specimens with a diameter of 1 to 3 mm were harvested. Samples were taken with a dedicated bioptome (Biopsy Forceps, Cordis Corporation) advanced through 9 French venous sheaths as described recently [20]. Samples were fixed under sterile conditions in 4% formaldehyde for light microscopy using hematoxylin and eosin (HE), Masson’s trichrome, picrosirius red, Giemsa and, Kongo red staining [14, 24, 26]. An avidin-biotin-immunoperoxidase method (Vectastain Elite ABC Kit, Vector, Burlingame, Calif) was used for immunohistochemistry comprising the following monoclonal antibodies to identify, localize and characterize mononuclear cell infiltrates: CD3 for T-cells (Novocastra Laboratories, Newcastle, UK), CD68 for macrophages (DAKO, Glostrup, Denmark), and HLA-DR-α (DAKO, Hamburg, Germany) to assess major histocompatibility complex (MHC) class II expression in antigen-presenting immune cells. Analysis of cardiac inflammation and fibrosis was determined by the cardiopathologist using established methods [24, 26]. Molecular detection of viral genomes within the myocardium was performed by nested (RT-) PCR (Ambion, Foster City, Calif) as described [12, 14, 27, 28].

### Electrophysiological evaluation by programmed ventricular stimulation for clinical risk assessment

PVS was performed from the right ventricular apex. Anti-arrhythmic drugs were discontinued for ≥2 days. Programmed electric stimulation was performed before sedation and opioid administration if necessary during procedure. The stimulation protocol consisted of two drive cycle lengths (600 ms and 400 ms) with one to three ventricular extrastimuli (≥200 ms) from the right ventricular apex. Inducibility was defined as sustained monomorphic or polymorphic VT (>30 s or requiring termination because of hemodynamic compromise) or unstable VT/VF leading to syncope and requiring early cardioversion/defibrillation as described recently [20].

### Morphologies of ventricular tachycardia

All induced sustained monomorphic VTs were categorized as right bundle-branch block—like or left bundle-branch block—like morphology (defined as predominant R or S in V1), inferior or superior axis (predominant R or S in aVF), left or right axis (predominant R or S in I), and precordial transition (first lead with a predominant R or S for left bundle-branch block and right bundle-branch block VTs, respectively).

### Assessment of cardiac function and morphology by contrast-enhanced cardiac magnetic resonance imaging

Contrast-enhanced cardiac MRI was performed on a 1.5 Tesla (T) scanner (Siemens Medical Systems, Germany) providing a gradient strength of 40 mT/m and maximum slew rate of 200 mT/m/msec. An advanced cardiac software package was used. Images were acquired with the subject in the supine position, by applying electrocardiographically gated breath-hold sequences as described before [15]. For LGE imaging a two-dimensional inversion-recovery segmented k-space gradient-echo MR sequence was performed using 0.15 mmol gadobutrol per kilogram of body weight (Gadovist, Bayer Healthcare, Germany) [15]. Two experienced investigators independently reviewed the image loops of each subject in a random fashion. For LGE image analysis both investigators visually judged the occurrence (presence versus absence), localization, and pattern of LGE [10, 29].

### Statistical analysis

Continuous variables are expressed as mean ± standard deviation and were compared using the student’s t-test. Categorical data are presented as proportions and were analyzed by chi-square or Fisher’s exact test. For this analysis, continuous variables were dichotomized using the patients`median as cut-off values or were dichotomized at established cut-off values where applicable. Comparison of two groups was done by t-test. To compare more than two groups ANOVA analysis was performed. Survival curves of patients grouped by pre-specified variables were calculated by Kaplan-Meier analyses and compared using log-rank test. Time point for begin of survival analysis was the date of EMB. Comparisons were considered statistically significant, if the two-sided p-value was ≤0.05. Statistical analyses were performed using SPSS software version 22.0 (SPSS Inc., Chicago, IL, USA).

## Results

### Patient population, clinical risk parameters and biomarkers

We retrospectively studied a cohort of n = 56 patients with impaired LVEF<50% due to NIDCM and suspected elevated risk for ventricular arrhythmia or sudden cardiac death between June 2009 and July 2013. Demographic details and baseline characteristics are presented in Table 1. Patients showed a mean age of 61.5±11.9 years, 46 (82.1%) of them were men. Cardiac and inflammatory biomarkers were increased throughout the cohort with mean BNP of 2135.3±2191.0 ng/l, mean TnI of 0.16±0.51 μg/l, mean CK of 122.7±112.6 U/l, and mean CRP of 1.7±4.5 mg/dl. Patients enrolled in the study showed an impaired LVF with a mean LVEF of 36.6±11.4% in cardiac MRI and 35.7±10.5% in TTE.

**Table 1:** Patients’ demographics and baseline characteristics.

All Patients	N = 56
***Clinical characteristics***	
Mean age, y ± SD	61.5±11.9
Gender, male	46 (82.1%)
BMI	26.6±4.1
NYHA functional class ≥ III	10 (17.9%)
NYHA functional class > II	19 (33.9%)
*Cardiac medication*	
ß-blockers	44 (78.6%)
ACE-inhibitors	43 (76.8%)
AT1-antagonists	6 (10.7%)
Diuretics	29 (51.8%)
Aldosterone antagonists	36 (64.3%)
*Echocardiography*	
LVEF (%)	35.7±10.5
LVEDD (mm)	57.7±7.9
Systolic PAP (mmHg)	32.4.1±11.4
*Cardiac biomarkers*	
BNP (ng/l)	2135.3±2191.0
TnI (μg/l)	0.16±0.51
CK (U/l)	122.7±112.6
CRP (mg/dl)	1.7±4.5
*Cardiac MRI*	
LVEF (%)	36.6±11.4
Positive LGE	35 (62.5%)
Edema	1 (1.8%)
***Results of endomyocardial biopsies***	
*Myocardial fibrosis in histology*	
Fibrosis	18 (32.1%)
- Mild fibrosis	8 (14.3%)
- Moderate to severe fibrosis	10 (17.9%)
*Immunohistology*	
CD3	16 (28.6%)
CD68	31 (55.4%)
MHC II	30 (53.6%)
*Virus detection in myocardium*	
Total Virus positive	21 (37.5%)
- PVB19	6 (28.6%)
- CVB3	5 (23.8%)
- EBV	5 (23.8%)
- HHV 6	5 (23.8%)
*Results of electrophysiological study*	
*Ventricular tachycardia or fibrillation*	
VT/VF inducible	10 (17.9%)

Values are n (%) or mean±standard deviation. ACE inhibitors—angiotensin converting enzyme, AT1-antagonists—angiotensin II type 1 receptor antagonist, BMI—body mass index, BNP—b-type natriuretic peptide (normal value < 100ng/l), CD—cluster of differentiation, CRP—C-reactive protein (normal value < 0.5mg/dl), CK—creatine kinase (normal value < 190U/l), CVB3 –Coxsackie virus B3, EBV—Epstein-Barr virus, HHV 6 –Human herpesvirus 6, l—liters, LGE—late gadolinium enhancement, LVEDD—left ventricular enddiastolic diameter, LVEF—left ventricular ejection fraction, MHC II—major histocompatibility complex class II, mmHg—millimeter of mercury, μg—micrograms, μl—microliters, n—number, ng—nanograms, NYHA—New York Heart Association, PAP—pulmonary artery pressure, in echocardiography, PVB19 –Parvovirus B19, SD—standard deviation, TnI—troponin I (normal value < 0.03μg/l), U—units, VT—sustained ventricular tachycardia, VF—ventricular fibrillation, y—years. Continuous variables were compared using t- test, categorical data were analyzed by chi-square test.

### Ventricular inducibility is not associated with histological and immunohistochemical markers of myocardial inflammation and fibrosis in endomyocardial biopsy

EMB revealed cardiac fibrosis in 18 (32.1%) patients, while immunohistochemistry detected 31 (55.4%) patients to be positive for CD68, 30 (53.6%) for MHCII, and 16 (28.6%) for CD3 as inflammatory markers. All patients underwent right-ventricular programmed stimulation with up to triple extra stimuli. VT/VF as defined above was induced in ten (17.9%) patients during PVS. Monomorphic VT was inducible in 7/10 (70%) patients, while 2/10 (20%) patients showed polymorphic VT. One patient (10%) presented with VF during PVS.

**Fig 1:**
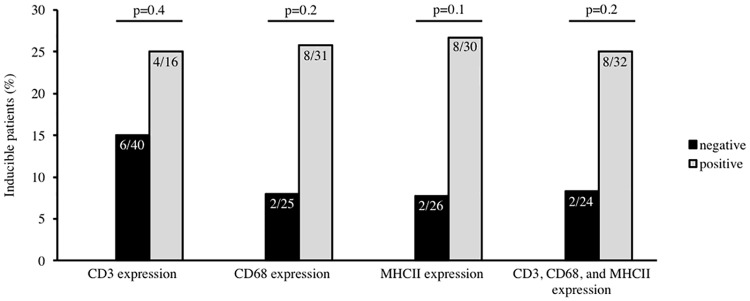
Association between ventricular inducibility and immunohistochemical markers of inflammation detected in endomyocardial biopsy. There was no significant association between the presence of the inflammatory marker CD3 and ventricular inducibility (p = 0.4), CD68 and ventricular inducibility (p = 0.2), MHCII and ventricular inducibility (p = 0.1), and the combination of all inflammatory markers (CD3, CD68, and MHCII) within the myocardium and ventricular inducibility (p = 0.2).

Fig 1 depicts the association between the inflammatory markers CD68, MHCII and CD3 revealed in immunohistochemistry and inducibility of ventricular arrhythmias during PVS. There was no significant association between each single inflammatory marker as well as their combination and ventricular inducibility (CD68: p = 0.2, MHCII: p = 0.1, and CD3: p = 0.4, combination of all three markers: p = 0.2, respectively, Fig 1).

**Fig 2:**
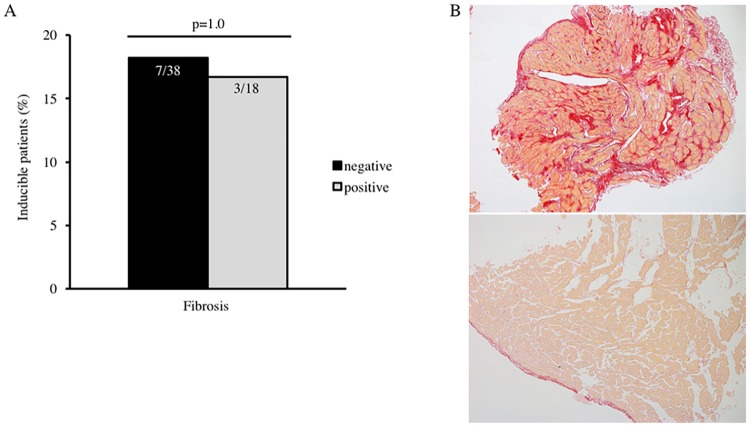
Association between ventricular inducibility and myocardial fibrosis detected in endomyocardial biopsy. **A.** There was no association between the presence and amount of myocardial fibrosis described in histopathology and ventricular inducibility during programmed ventricular stimulation (p = 1.00). **B.** The upper panel of Fig 2B illustrates an example of severe myocardial fibrosis in endomyocardial biopsy detected by picrosirius red staining in a patient with non-ischemic dilated cardiomyopathy and no inducible ventricular arrhythmias. The lower panel depicts the histology of healthy myocardium with a fibrosis area of <3%.

Similarly, we could not find a correlation between the presence of myocardial fibrosis described in histopathology and inducibility of VT or VF during stimulation, presented in Fig 2A (p = 1.00). The upper panel of Fig 2B illustrates exemplary the myocardium of a patient with severe myocardial fibrosis in EMB detected by picrosirius red staining, but no inducible ventricular arrhythmias during PVS. The lower panel depicts the histology of healthy myocardium with a fibrosis area of <3%.

### Ventricular inducibility is associated with late gadolinium enhancement in cardiac MRI

**Fig 3:**
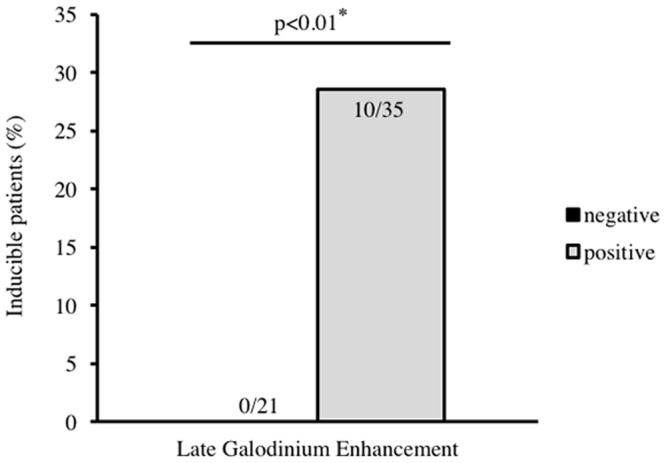
Association of ventricular inducibility with late gadonlinium enhancement in contrast-enhanced cardiac MRI. Fig 3 depicts the association of ventricular inducibility in patients with non-ischemic cardiomyopathy and the detection of positive late gadolinium enhancement in contrast-enhanced cardiac MRI. Ventricular inducibility correlates significantly with the presence of late gadolinium enhancement, p<0.01.

Contrast-enhanced cardiac MRI detected positive LGE in 35 (62.5%) patients, one (1.8%) patient showed intracardiac edema. The occurrence of hemodynamically relevant VT or VF during PVS correlated significantly with the presence of LGE in cardiac MRI as shown in Fig 3 (p<0.01). In 10/35 (28.6%) of the LGE positive-group either monomorphic, sustained VT (n = 7), polymorphic VT causing syncope (n = 2), or VF (n = 1) was inducible. Thus, no sustained ventricular arrhythmia requiring cardioversion or defibrillation could be induced in patients without LGE in cardiac MRI (0/21). The absence of LGE in MRI precludes inducibility of ventricular arrhythmias in these patients. However, LGE was not predictive in detection of clinical arrhythmic events (data not shown).

**Fig 4:**
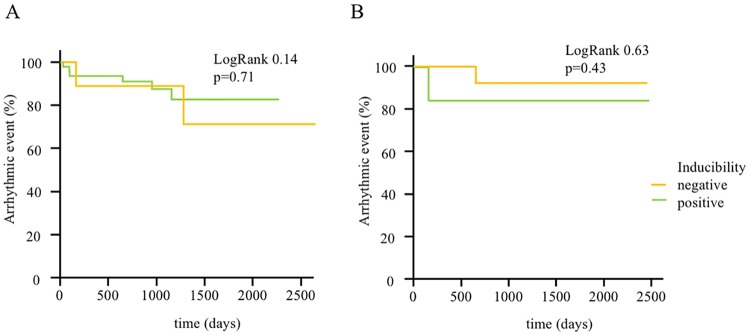
Kaplan-Meier-Curves for the occurrence of primary endpoints stratified by inducibility in programmed ventricular stimulation in all patients (A) and in a subgroup of patients with low inflammatory activity (B). **A.** Kaplan-Meier estimates illustrate the occurrence of the primary endpoints hemodynamically relevant sustained ventricular tachycardia and/or adequate ICD-therapy (antitachycardia pacing, shock) for all enrolled patients during follow-up. There is no significant difference between inducible and non-inducible patients regarding the occurrence of ventricular arrhythmia (LogRank 0.14, p<0.71). **B.** Kaplan-Meier estimates illustrate the occurrence of the primary endpoints hemodynamically relevant sustained ventricular tachycardia and/or adequate ICD-therapy (antitachycardia pacing, shock) during follow-up for a subgroup of patients with low inflammatory activity. There is no significant difference between inducible and non-inducible patients regarding the occurrence of ventricular arrhythmia (LogRank 0.63, p<0.43).

Accordingly, inducibility of VTs or VF was not predictive for arrythmogenic events in our cohort (Fig 4A). During a mean follow-up of 2.6 years, 9 (16.1%) patients reached the primary endpoint. In 6/9 (66.7%) patients monomorphic VTs were found and terminated by antitachycardia pacing therapy (ATP). 1/9 (11.1%) patient with sustained, polymorphic VT and 2/9 (22.2%) patients with VF received adequate therapy by an ICD-shock which terminated the ventricular arrhythmia.

In recent studies, PVS was not a clear predictor of arrhythmic events in patients with inflammatory, dilative cardiomyopathy. We hypothesized that this could be ascribed to the ongoing remodeling processes leading to novel arrhythmogenic substrates. Thus, we analyzed the value of PVS in a subgroup of patients with markers of low inflammation and remodeling in EMB. There was no significant difference over a follow up period of 2.6 years. However, no patient in the non-inducible-group suffered from an arrhythmogenic event within one year (Fig 4B), which may indicate that persistant, ongoing inflammatory remodeling processes lead to novel arrhythmogenic substrates in patients with NIDCM.

## Discussion

In our present study we evaluated the correlation of positive LGE in cardiac MRI, the inflammatory degree and presence of fibrosis in EMB with the inducibility of ventricular arrhythmias during PVS in patients with NIDCM. Inducibility comprised the induction of VT and VF to assess the overall life-threatening arrhythmogenic risk in contrast to induction of VT alone.

To our knowledge, this is the first study to compare these findings in a broader context, in particular linking histopathology and immunohistochemistry of EMB to the results of PVS.

Our current findings show that the presence of LGE in contrast-enhanced cardiac MRI correlates significantly with the inducibility of VTs during PVS in patients with NIDCM. Our findings are in line with several other studies that described this correlation previously [8, 11]. The absence of LGE is an excellent negative predictor for the inducibility of ventricular arrhythmias in our cohort. However, inducibility of ventricular arrhythmias was not predictive for clinical arrhythmic events in our cohort, which may be due to the limited sample size and follow-up time in our study.

It is not completely clear, why PVS is a much better predictor in patients with ischemic compared to non-ischemic cardiomyopathy. One reason could be that in ischemic cardiomyopathy the remodeling process is completed resulting in myocardial fibrosis and in a definite myocardial scar in contrast to chronic inflammatory heart disease, in which the cardiac remodeling is an ongoing and progressive process. Interestingly, we found no patient with low markers of remodeling and negative programmed stimulation suffering from an arrhythmic endpoint within one year. However, during the complete follow up period, there was no significant difference in the subgroup of patients with low inflammation reflecting the problem of arrhythmic event prediction in a disease with ongoing remodeling.

Our results highlight the advantages of contrast-enhanced cardiac MRI as a diagnostic tool to detect myocardial fibrosis. Furthermore, we show that PVS can be part of a clinical work-up based on individualized criterions to further characterize patients with an history of arrhythmias, even if the inducibility itself did not correlate with the occurrence of our defined study endpoints over a long-time period [23]. Our study may suggest the hypothesis that the absence of LGE identifies patients who have a lower risk for arrhythmogenic events irrespective of the indication for an ICD implantation.

In this regard, it was argued that cardiac regions showing positive LGE in MRI could also represent arrhythmogenic areas as origin of VTs in patients with NIDCM [19, 20]. Therefore, electrophysiological analysis employing PVS along with contrast-enhanced MRI may serve as helpful diagnostic tool in selected patients to assess their individual risk for sudden cardiac death [17, 21, 23]. Deeper evaluation is possible by electroanatomical mapping during eletrophysiological examinations, which may also help to characterize arrhythmogenic sites within the ventricle to enable an ablative therapy of ventricular arrhythmias if necessary during the course of the disease [5, 19, 20].

One has to keep in mind that NIDCM is a chronically progressive disease finally leading to HF and concomitant complications. The goal is to identify patients at risk at an early stage of the disease to prevent life-threatening events. Thus, inducibility during PVS can only represent a “snapshot” and momentary appraisal of the disease and is of limited predictive value for future arrhythmic events depending on severity and stage of the underlying NIDCM. That is also reflected by our observation that inducibility during PVS was not correlated with adverse clinical outcome defined as occurrence of relevant ventricular arrhythmogenicity.

In line with other groups, we reported previously that detection of myocardial inflammation in EMB seems to be an excellent predictor of prognosis for adverse events during the course of the disease [14, 30].

Surprisingly, we could not find an association between the inducibility of ventricular arrhythmias and myocardial fibrosis or inflammation detected in undirected right-ventricular EMB. Therefore, this observation is of great interest and might suggest that undirected routine EMB is not a useful tool in arrhythmogenic risk stratification of patients with NIDCM.

In our patient collective, stainings of EMB revealing infiltrating inflammatory cells and fibrosis within the myocardium could not be linked to the inducibility of relevant ventricular arrhythmia during PVS and therefore fails to serve as efficient prognostic and diagnostic tool to identify patients at risk for VTs and sudden cardiac death. As this study is the first to describe that there is no correlation between biopsy results and findings during PVS, larger randomized studies are needed to confirm our first observations.

There are several reasons why EMB may fail to predict inducibility: Fibrosis detected in MRI is often located in the midventricular area of the ventricle wall and biopsy specimens are taken from the endomyocardium. Hence, the area of intial cardiac remodeling may not be reflected in EMB. In particular, classical EMB is not targeted to a specific, pre-defined region of the ventricle and therefore might not identify pathological areas within the myocardium that represent arrhythmogenic substrate. Thus, the sample error rate in routine EMB could be rather high [20]. This observation is supported by the fact that there was a clear correlation of fibrosis, inflammation and arrhythmia in a patient who was biopsied in low voltage areas. Here, we could show that a site-directed biopsy visualizing the bioptome tip in an electroanatomical mapping system is feasible and offers additional possibilities for the investigation of low voltage areas in arrhythmogenic cardiomyopathies including myocarditis [31].

Since the detection of LGE could generally be observed in progressed stages of NIDCM, the absence of LGE does not exclude earlier stages of NIDCM, which can proceed and result in terminal HF and arrhythmogenic events [10, 20]. However, it is tempting to speculate that the exclusion of LGE, myocardial inflammation and/or fibrosis and non-inducibility can predict a favorable prognosis of patients.

We are aware that our findings are rather observational and hypothesis-generating, as we retrospectively evaluated 56 patients with impaired LVF due to NIDCM. Therfore, further large-scale prospective and randomized studies are needed to evaluate the benefit of an additional risk assessment with PVS in patients with NIDCM, along with endomyocardial biopsy and cardiac MRI regarding arrhythmogenic risk and appropriate ICD implantation.

## Conclusion

Our findings suggest that the presence of LGE identifies patients at risk for sudden cardiac death suffering from NIDCM and may be a valuable additional diagnostic tool for risk stratification in these patients. Ventricular inducibilty in PVS is associated with the presence of LGE in cardiac MRI, but not with the presence or the degree of myocardial fibrosis or inflammation in undirected EMB. Inducibility in PVS could not predict future relevant VTs or VF during follow-up. Therefore, the diagnostic and prognostic impact of routine biopsy findings and PVS alone is insufficient to identify patients at risk for recurrent arrhythmic events in our observed cohort.

## Abbreviations

ATP: antitachycardia pacing
BNP: B-type natriuretic peptide
CK: creatine kinase
CRP: C-reactive protein
EMB: endomyocardial biopsy
HE: hematoxylin and eosin
HF: heart failure
ICD: implantable cardioverter-defibrillator
LGE: late gadolinium enhancement
LVEF: left ventricular ejection fraction
LVF: left ventricular funtion
MHCII: major histocompatibility complex class II
MRI: magnetic resonance imaging
NIDCM: non-ischemic dilative cardiomyopathy
NYHA: New York Heart Association functional class
PV: predictive value
PVS: programmed ventricular stimulation
TnI: troponin I
VF: ventricular fibrillation
VT: ventricular tachycardia
